# One-year real-life outcomes of lanadelumab therapy in Romanian patients with hereditary angioedema due to C1-inhibitor deficiency

**DOI:** 10.3389/falgy.2025.1636425

**Published:** 2025-08-21

**Authors:** Noémi Anna Bara, Valentin Nădășan, Diana Deleanu

**Affiliations:** ^1^ Romanian Angioedema Center of Reference and Excellence, Centrul Clinic MediQuest, Sangeorgiu de Mures, Romania; ^2^ Internal Medicine, George Emil Palade University of Medicine, Pharmacy, Science, and Technology of Targu Mures, Targu Mures, Romania; ^3^ Hygiene Department, George Emil Palade University of Medicine, Pharmacy, Science, and Technology of Targu Mures, Targu Mures, Romania; ^4^ Allergology Department, “Professor Doctor Octavian Fodor” Regional Institute of Gastroenterology and Hepatology Cluj-Napoca, Cluj Napoca, Romania

**Keywords:** hereditary angioedema, C1-inhibitor, treatment, outcome, lanadelumab

## Abstract

**Introduction:**

In the majority of patients with hereditary angioedema (HAE) due to C1-inhibitor deficiency (HAE-C1INH), effective long-term prophylactic (LTP) treatment can achieve complete disease control. Lanadelumab is one of the first-line option recommended for this purpose. Our study aimed to evaluate changes in disease control, quality of life, and attack frequency among Romanian HAE-C1INH patients, during the first year of treatment with lanadelumab.

**Methods:**

This non-interventional prospective study included the Romanian HAE-C1INH patients enrolled in the first year of the national lanadelumab treatment program. Angioedema Control Test, (AECT), Angioedema Quality of Life Questionnaire (AE-QoL) and attacks frequency were recorded at baseline and at 3, 6, 9 and 12 months.

**Results:**

Twenty-four patients (14 women [58.3%], 10 men [41.7%]) initiated lanadelumab therapy, with a mean age of 40.7 years. Most had HAE-C1INH type I (22 patients, 91.7%), and one patient was under 18 years of age. Ten patients switched from LTP with intravenous plasma-derived C1-INH, while 14 were previously managed with on-demand therapy only. Baseline scores were: AECT 4.5 [interquartile range (IQR) 2.0], AE-QoL 66.1 [standard deviation (SD) 18.3], and a mean attack frequency of 10.0 (IQR 4.0) (over the preceding three months). Improvements were observed at each follow-up point, with respective scores at 3, 6, 9 and 12 months as follows: three months: AECT 12.0 (IQR 5.8) / AE-QoL 35.3 (SD 23.2)/ attacks 3.4 (IQR 5.0); six months: AECT 12.3 (IQR 5.3) / AE-QoL 35.4 (SD 25.4)/ attacks 2.8 (IQR 4.8); nine months: AECT 12.6 (IQR 5.8) / AE-QoL 34.1 (SD 23.2)/ attacks 2.2 (IQR 3.8) and 12 months: AECT 12.9 (IQR 5.5) / AE-QoL 32.1 (SD 21.6) / attacks 1.4 (IQR 2.0). Seven patients became symptom-free after the first dose, and four more achieved this status within the first three months.

**Discussion:**

LTP with lanadelumab provided effective disease control and significantly improved quality of life in patients with HAE-C1INH over the course of one year. Regular evaluations at relatively short intervals by the availability and ease of administration of validated questionnaires serve as a useful tool for clinicians in the comprehensive assessment of HAE patients and offer a valuable means of monitoring treatment effectiveness.

## Introduction

Hereditary angioedema (HAE) is a rare, genetic disorder, attributed in most cases to the *SERPING 1* gene mutations, resulting in functional (HAE-C1INH type1) or quantitative (HAE-C1INH type2) deficiency of the C1 esterase inhibitor (C1INH) protein (1, 2).

The disease follows an autosomal dominant inheritance pattern, with *de novo* mutations accounting for approximately 25% of cases (2, 3).

The prevalence of the disease is estimated at approximately 1 in 50,000 individuals, with no significant differences observed across sex or ethnic groups (2, 4).

Clinical symptoms consist in recurrent painful swellings of the subcutaneous and/or submucosal tissues, mediated by the vasoactive peptide, bradykinin (5)*.* The swelling can affect any body part, with the skin, gastrointestinal tract, and upper airways being the most commonly involved sites (2, 4).

If untreated, the attacks last 2–5 days (2, 3, 6, 7) and in case of upper airway edema it can be life-threatening due to risk of asphyxiation (2, 8).

Similar clinical pictures are found in the HAE forms with normal C1-INH level (HAE-nC1INH), which result from distinct genetic mutations. These includes variants in factor XII *(HAE-F12)* (9), plasminogen (HAE-*PLG*), angiopoietin (HAE-*ANGPT1*), kininogen (HAE-*KNG1*), myoferlin (HAE-*MYOF)* (2, 10–15) (HAE-HS3ST6) (16), (HAE-CPN) (17), or (HAE-DAB2IPE) (18) genes. Nevertheless, a substantial number of HAE-nC1INH cases remain genetically unexplained (HAE-UNK) (19).

Although certain trigger factors have been identified, HAE attacks are often unpredictable and can be intensely painful, significantly affecting patients' daily functioning, including work and school productivity (1, 2, 20–27).

In a survey conducted among Romanian HAE-C1INH patients, the mean number of missed work or school days was 9.3 over the 12-month evaluation period (28). As such, treatment primarily aims to enhance the patient's quality of life and prevent the occurrence of attacks, ultimately seeking to achieve complete disease control (19). In most cases, this goal can be attained through the regular administration of specific medications, referred to as long-term prophylaxis (LTP).

At present, three medications are approved as first-line therapy for LTP: the plasma derived C1INH, the kallikrein inhibitor monoclonal antibody—lanadelumab and the small molecule plasma kallikrein inhibitor berotralstat. Attenuated androgens are considered second-line treatment drugs in HAE -LTP and the antifibrinolytics, such as tranexamic acid, are no longer recommended (19).

In accordance with EAACI/WAO guideline recommendations, the initiation of LTP should be guided by an assessment of disease activity, the impact on the patient's quality of life, the availability of healthcare resources, insufficient response to on-demand therapy, and the patient's informed preferences (19).

These parameters can be assessed using patient-reported outcomes (PROs), such as the Angioedema Control Test (AECT), the Angioedema Quality of Life Questionnaire (AE-QoL), and the attack diary. These tools are also valuable for evaluating the safety and efficacy of chronic treatments. In this manner, a personalized and comprehensive treatment plan can be developed for each patient with HAE.

As an angioedema attack can appear during the LTP, the on-demand therapy (pdC1INH, rhC1INH icatibant and ecallantide) should be available (19, 29), or, if these are not accessible, fresh frozen plasma can be administered (19, 29).

Our study aimed to assess the changes of quality of life, disease control and attacks frequency in the Romanian HAE-C1INH patients during the first year of treatment with the kallikrein inhibitor monoclonal antibody—lanadelumab, based on the above-mentioned PRƠ's.

## Material and methods

This was a non-interventional study of the HAE-C1INH patients from Romania included in the first year of lanadelumab treatment program, conducted by the Romanian HAE Center of Reference and Excellence. Patients were announced about the availability of this drug via an on-line meeting when the inclusion criteria were also presented: age of ≥12 years, confirmed diagnosis of HAE-C1INH type1 or 2, and for the last 3 months period: 1. the frequency and severity of the attacks (≥2 attacks/last 3 months based on patient diary),and/or 2. an inadequate control of the disease on used treatment (AECT score <10), and/or 3. the impact of the disease on quality of life (based on AE-QoL questionnaire, score ≥39, moderate or severe effect), and/or 4. an important burden of the used treatment and 5. the patients preferences.

Those who did not attend this meeting were informed at the annual visit (face-to-face visit or phone-call visit).

At pre-treatment visit (T0) the mentioned questionnaires were completed electronically, after a phone-call discussion or during the annual visit at the Center. Patients included in the treatment program were evaluated using the same tools, at every three months (T1, T2, T3 and T4).

Participation in the treatment program was independent of the patients' consent to participate in this survey.

Both the AECT and the AE-QoL are available in Romanian. While not specific to HAE, these tools have been validated and are frequently used in the context of this disorder, both in clinical trials and routine clinical practice, due to their brevity and ease of scoring.

AECT is a four-item patient-related tool used in subjects with recurrent angioedema. The first question refers to the attack's frequency and the second one to their consequences on the patient's daily life. The unpredictability of the attacks is evaluated by the third question and, the last one assesses the used treatment efficiency (30, 31). Two versions for AECT are accessible: for a recall period of four weeks and three months respectively. In our study the last one was used.

AE-QoL contains 17 items grouped in four domains: functioning, fatigue/mood, fears/shame, and nutrition. In this case the questions are referred to the previous four weeks (2, 32).

In both cases answers are predetermined using a five-point verbal rating scale, and for all responses the score ranges from 0 to 4 points (33).

In the case of AECT the total score is 16 points, which reflects a complete control of the disease, the main goal of the treatment in HAE. A result of ≥10 points show a well-controlled disease and if it is <10 points, indicates an inadequately controlled status. The grades used for this questionnaire were the following: very often = 0 points, often = 1 point, sometimes = 2 points, seldom = 3 points, and not at all = 4 points Increasing the score by three points or more at two consecutive visits, can be considered as an important improvement for the patient (34).

The AE-QoL score is obtained by converting the sum of points for every question to a linear 0–100 scale with higher values pointing to a higher QoL impairment. Intervals of total scores of 0–23, 24–38, and 39 or more describe patients with “no effect,” “small effect,” and “moderate to large effect” of swelling episodes on their quality of life, respectively (14). In the case of this questionnaire, the minimal clinically important difference was established to be six points (35).

For this questionnaire the following grades were used: never = 0 point, seldom = 1 point, sometimes = 2 points, often = 3 points and very often = 4 points.

The four individual domain scores were computed by summing the points assigned to the specific questions to each dimension, as follows: functioning—questions 1, 2, 3 and 4, fatigue/mood questions 6, 7, 8, 9 and 10, fears/shame questions 12, 13, 14, 15, 16 and 17 and nutrition questions 5 and 11.

Attacks frequency and severity were established using the attacks diary. For every swelling episode the same data were recorded: date of the attack, location, severity (mild/moderate or severe depending on the capability to perform daily activities) and the used treatment. Patient utilized either the paper-and-pen diary or the electronic one.

Information regarding socio-demographic data (age, sex), HAE type, and used treatment, were collected from the Romanian HAE Registry.

The study was conducted in compliance with the requirements set forth in the Declaration of Helsinki and was approved in December 2022 by the IRB of the George Emil Palade University of Medicine, Pharmacy, Science, and Technology of Targu Mures, Romania (Decision no. UMFST-nr 1914/02.11.2022 -REG-74-F03-Ed.02).

### Data analysis

Descriptive statistics were calculated to summarize patient demographics and therapeutic management of HAE.

The normality of numerical variables was checked using the Kolmogorov–Smirnov test of normality.

Mean AECT and AE-QoL scores were calculated at three months intervals, and the Friedman test with pairwise post-test comparison were applied to compare values at each successive interval. Comparison between mean scores in men vs. women, and between patients on LTP vs. OD treatment were performed using Mann–Whitney or independent samples *t* test.

Two-sided *p*-values were calculated, and the threshold for statistical significance was set at 0.05. Statistical analyses were performed using the Statistical Package for the Social Sciences Software (version 22.0, SPSS, Chicago, IL).

## Results

### Study population

At the time of the study, 123 patients aged 12 years and older with HAE-C1INH were registered in the Romanian HAE Registry. Of these, 30 patients participated in an online informational meeting, and 93 received details about the lanadelumab treatment program during their annual clinical visit. A total of 24 patients, 14 women (58.3%) and 10 men (41.7%) were subsequently enrolled in the first year of the lanadelumab treatment program.

**Figure 1:**
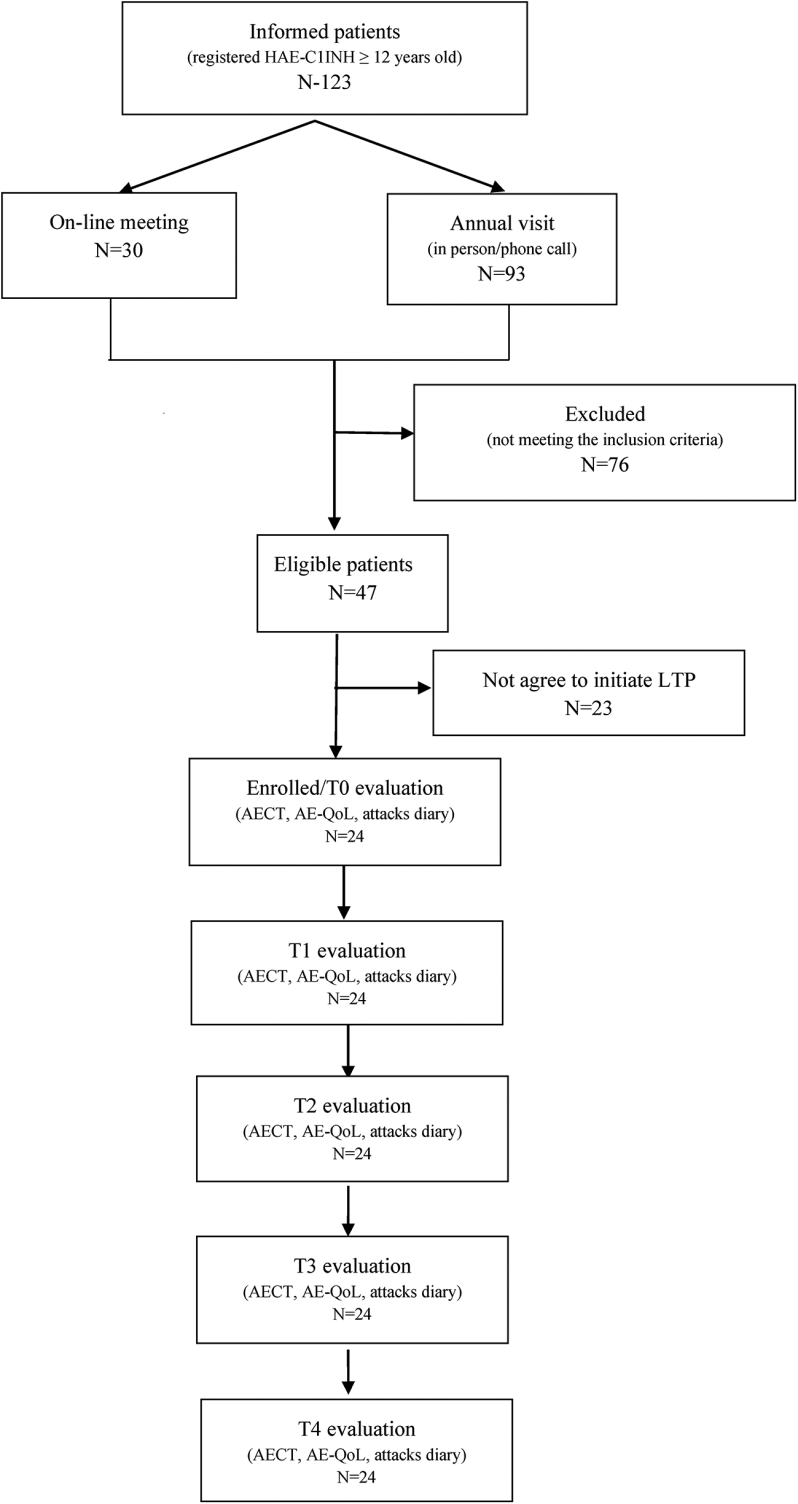
Study flow chart.

Numerical details about the study population, eligible patients, and final study sample are represented in Figure 1.

### Socio-demographic data

The mean age was 40.70 years (range 15–66 years), including one adolescent patient and one patient >65 years old. Twenty-two (91.66%) patients had HAE-C1INH type 1 and two (8.34%) HAE-C1INH type 2.

Ten patients used long term prophylaxis therapy with the intravenous form of pdC1INH administered twice per week in dose of 1,000 UI, and 14 only on-demand treatment with icatibant or pdC1-INH.

Co-morbidities were present in eight patients (33.34%). Cardiovascular disorders were most common, observed in four patients (including hypertension, ischemic heart disease, and dyslipidemia). Autoimmune conditions were identified in two patients (thyroiditis and psoriasis), while single cases of gastrointestinal disease (chronic gastritis), allergy (grass pollen rhinitis), and endocrine disorder (polycystic ovary syndrome) were also reported.

The sociodemographic data (sex, age, HAE type), specific treatment used at T0 evaluation and comorbidities are presented in Table 1.

**Table 1:** Socio-demographic data (sex, age, HAE type), specific treatment and co-morbidities at T0 evaluation.

Patient characteristics	Value
Sex	
Female	14 (58.3%)
Men	10 (41.7%)
Age (mean)	40.7
15–18	1 (4.2%)
19–45	14 (58.3%)
46–64	8 (33.3%)
≥65	1 (4.2%)
HAE type	
1	22 (91.66%)
2	2 (8.34%)
LTP	
Yes	10 (41.66)
No	14 (58.33)
Co-morbidities	
Yes	8 (33.34%)
No	16 (66.67%)

LTP, long-term prophylaxis.

### Lanadelumab treatment

Lanadelumab was administered in a dose of 300 mg every 2 weeks. In case of 6 months symptom-free interval the dose was reduced to 300 mg at 4 weeks. This was the case in seven patients (29.16%). In five (20.83%) cases, due to patients' preferences (fear to have an attack), the interval of administration was increased progressively with four days, up to 20/21 days (based on German experience, 36). Seven patients (29.16%) continued having attacks and they remained at the dose of 300 mg every two weeks. One patient increased the interval of administration to six weeks, and the symptom free status was maintained at the 12 month and 24-month evaluation too.

In all cases, the first two doses of lanadelumab were administered by medical personnel, and then by self-administration as home-treatment. Adverse effects were evaluated following the initial two doses and subsequently at each follow-up visit (T1, T2, T3, and T4).

In patients receiving LTP with pdC1INH, the switch to lanadelumab was made as soon as possible after the last dose of pdC1-INH.

### Attacks frequency

**Figure 2:**
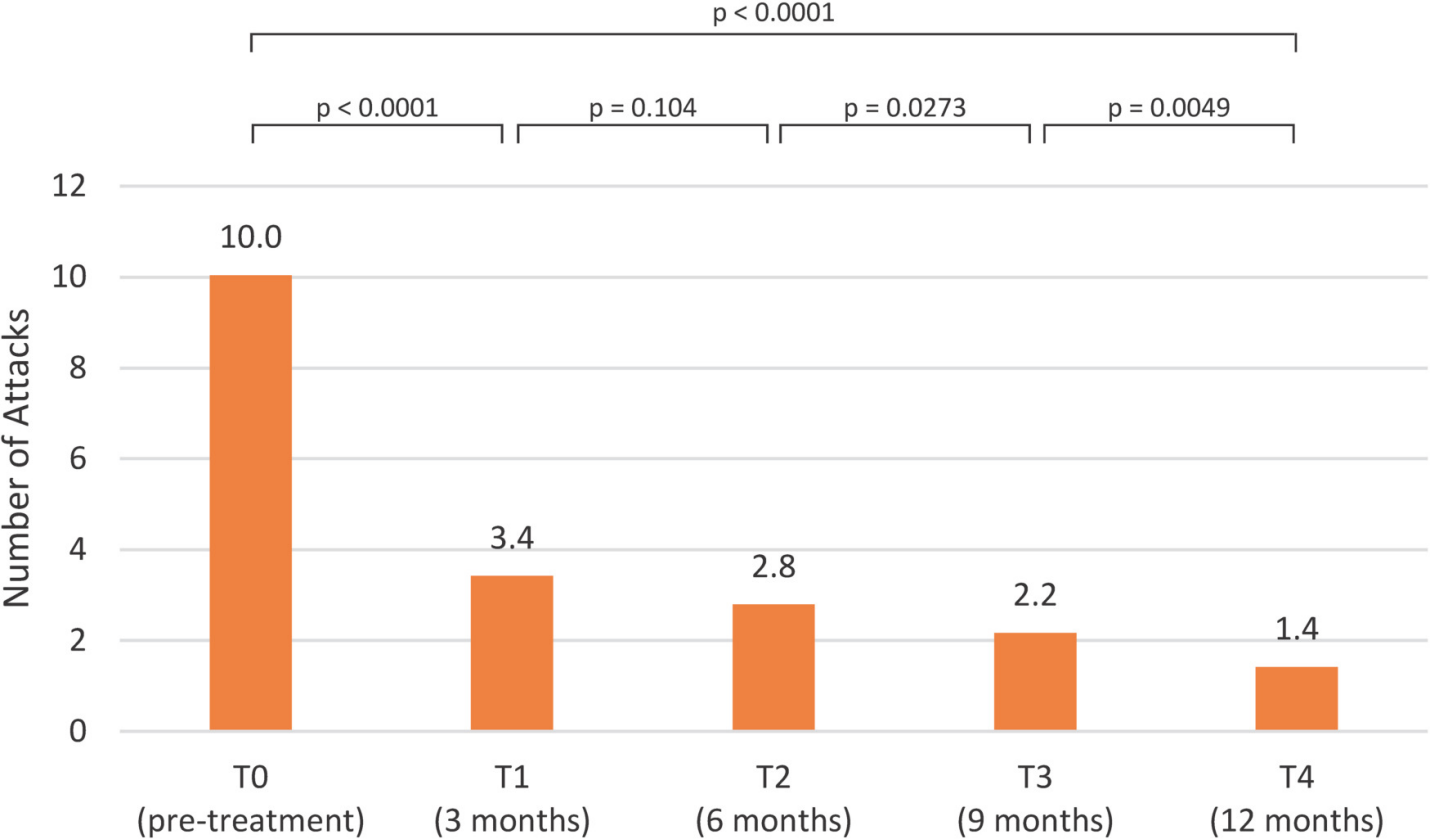
Attacks frequency pre-treatment and post-treatment visits.

The mean attack frequency was 10.0 [interquartile range (IQR) 4.0] at T0 visit, and decreased to 3,4 (IQR: 5.0) at T1assessment (*p* < 0.0001). Swelling episodes continued to reduce, being 2.8 (IQR: 4.8) at T2 (*p* = 0.104), 2.2 (IQR: 3.8) at T3 (*p* = 0.0273) and 1.4 (IQR: 2.0) at T4 evaluation (*p* = 0.0049) (Figure 2).

### Patient-related outcome measures

#### AECT score

At the pre-treatment evaluation, the mean AECT score was 4.5 (IQR: 2.0). With the exception of one patient, who had a controlled disease at baseline (AECT score = 10) and was already on LTP, all patients had scores below 10. In this particular case, the patient was switched to lanadelumab due to the burden associated with frequent intravenous injections.

**Figure 3:**
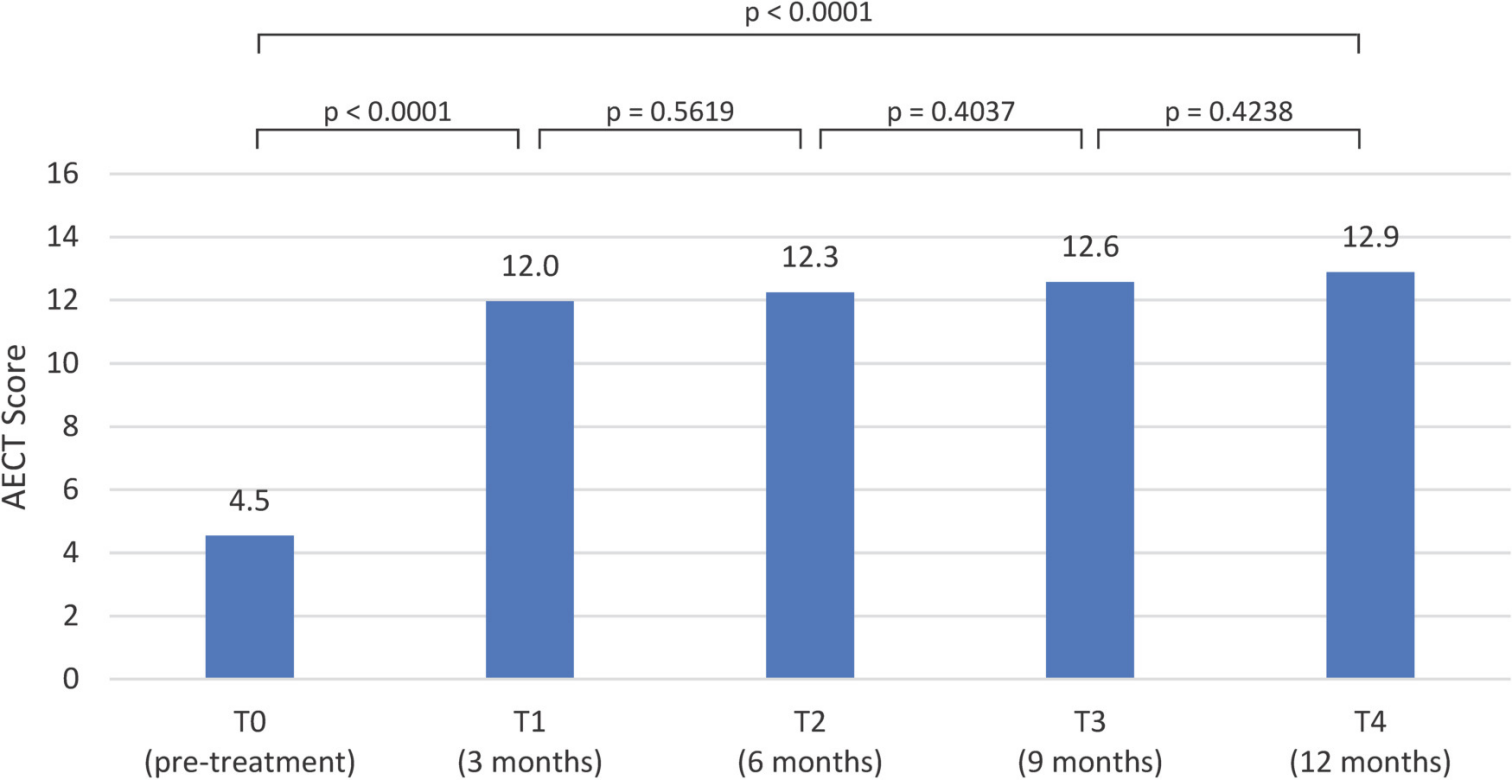
Mean AECT scores pre-treatment and post-treatment visits.

Following treatment initiation, the AECT scores showed a progressive improvement: the mean score at T1 was 12.0 (IQR: 5.8), at T2 it was 12.3 (IQR: 5.3), at T3 it increased to 12.6 (IQR: 5.8), and at T4 reached 12.9 (IQR: 5.5) (Figure 3).

### AE-QoL assessment

**Figure 4:**
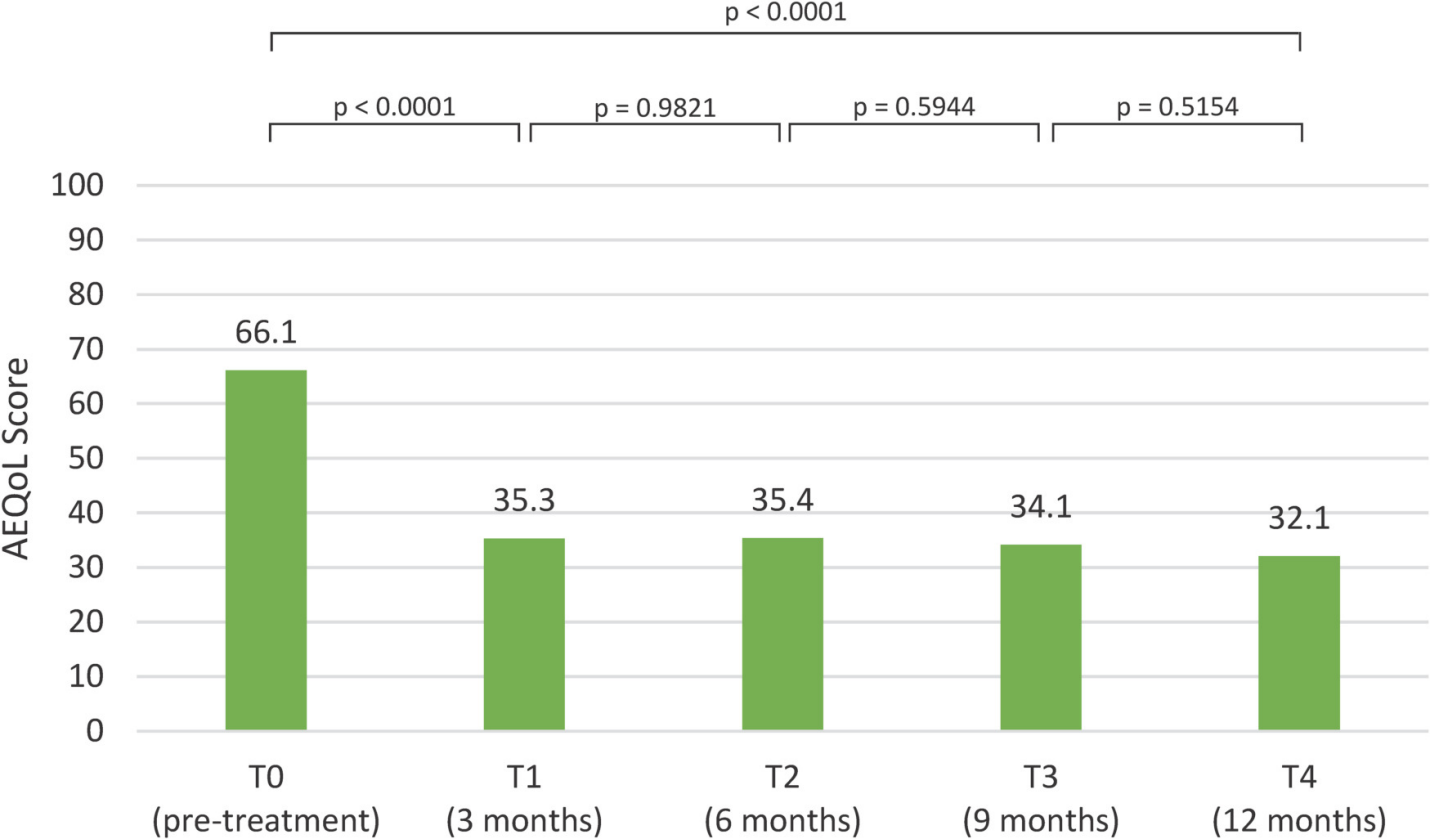
AE-QoL scores pre-treatment and post-treatment visits.

The mean AE-QoL total score at baseline, prior to lanadelumab initiation, was 66.1 [standard deviation (SD) 18.7], indicating a substantial impairment in quality of life. At the T1 assessment, this score significantly decreased to a mean of 35.3 (SD: 23.7). The improvement was sustained over time, with mean scores of 35.4 (SD: 25.9) at T2, 34.1 (SD 33.0) at T3, and 32.1 (SD: 22) at the T4 evaluation (Figure 4).

Regarding the AE-QoL domain scores, comparison of rank sum differences between baseline (T0) and the first follow-up (T1) demonstrated a statistically significant improvement (*p* < 0.0001) in the total score: 56, as well as across all domains: functioning 49.3, fatigue/mood 48.5, fears/shame 47.5, and nutrition 40. At later time points, slight deterioration was observed: in the total score (−5.5) and the fatigue/mood score (−2.0) between T1 and T2, and in the functioning score (−1.15) from T3 to T4. The nutrition domain showed a more pronounced decrease from T1 to T2 (−12.5). The fear/shame domain showed an improvement trend at all follow-up evaluations.

A summary of the AE-QoL total and domain-specific scores across all time points is presented in Table 2.

**Table 2:** AE-QoL total and domain scores.

AE-QoL score	Friedman test	Pairwise comparison	Rank sum difference	*P*-value
Total	Fr = 53.769 *p* < 0.0001	T0–T1	56	<0.0001
		T1–T2	−5.5	>0.05
		T2–T3	12	>0.05
		T3–T4	3.5	>0.05
Functioning domain	Fr = 59.074 *p* < 0.0001	T0–T1	49.33	<0.001
		T1–T2	2.54	>0.05
		T2–T3	0.13	>0.05
		T3–T4	−1.15	>0.05
Fatigue/Mood domain	Fr = 39.012 *p* < 0.0001	T0–T1	48.5	<0.001
		T1–T2	−2	>0.05
		T2–T3	1	>0.05
		T3–T4	7.5	>0.05
Fears/Shame domain	Fr = 42.922 *p* < 0.0001	T0–T1	47.5	<0.001
		T1–T2	4	>0.05
		T2–T3	3	>0.05
		T3–T4	4.5	>0.05
Nutrition domain	Fr = 25.947 *p* < 0.0001	T0–T1	40	<0.001
		T1–T2	−12.5	>0.05
		T2–T3	10.5	>0.05
		T3–T4	4	>0.05

When comparing quality-of-life impairment between women and men, the following results were observed: at the T0 evaluation, the mean total AE-QoL score was 73.79 in women vs. 55.40 in men. Domain-specific mean scores at T0 were: functioning—76.57/58.20, fatigue/mood—67.86/47.50, fears/shame—83.07/62.60, and nutrition—57.43/65.20 (women/men). By the T4 visit, these scores had improved, with the mean total score decreasing to 34.29 in women and 29.00 in men. Corresponding domain scores at T4 were: functioning—19.29/23.20, fatigue/mood—43.57/26.00, fears/shame—42.50/37.60, and nutrition—24.71/35.20 (women/men). A full summary of these results, including data from T2 and T3 assessments, is provided in Table 3.

**Table 3:** AE-QoL comparison by sex (F-female, M-male).

AE-QoL	T0	*p*-Value	T1	*p*-Value	T2	*p*-Value	T3	*p*-Value	T4	*p*-Value
Total										
F	73.79	0.0303	41.93	0.1138	41.64	0.1688	38.21	0.4464	34.29	0.558
M	55.40		26.10		26.60		28.40		29.00	
Functioning domain										
F	76.57	0.0375	27.07	0.0936	23.21	0.6377	23.64	0.7461	19.29	0.7017
M	58.20		10.70		14.60		12.60		23.20	
Fatigue/mood domain										
F	67.86	0.1875	44.43	0.1349	48.43	0.0531	44.71	0.4121	43.57	0.2186
M	47.50		28.50		22.50		31.50		26.00	
Fears/shame domain										
F	83.07	0.0059	53.43	0.2412	51.14	0.2533	49.07	0.3955	42.50	0.8147
M	62.60		39.50		36.10		37.60		37.60	
Nutrition domain										
F	57.43	0.5178	33.29	0.6381	37.71	0.6382	33.21	0.9298	24.71	0.5571
M	65.20		25.20		32.60		31.40		35.20	

### Comparison of AECT, AE-QoL and attacks frequency regarding previously used treatment

At the T0 evaluation, patients previously managed with on-demand treatment showed mean values of 3.7 (AECT), 68.9 (AE-QoL), and 11.1 (attack frequency), compared to 5.9, 61.6, and 8.2 respectively, in patients already receiving long-term prophylaxis (LTP). At the T1 visit, these values improved to 12.1/11.7 (AECT), 30.8/38.7 (AE-QoL), and 3.1/3.9 (attack frequency) in the on-demand and LTP groups, respectively. By the T4 assessment, values further improved to 13.2/12.3 (AECT), 28.9/37.4 (AE-QoL), and 1.4/1.4 (attack frequency). A detailed summary of outcomes across all time points, including T2 and T3 evaluations, is provided in Table 4.

**Table 4:** Comparison of AECT, AE-QoL and attacks frequency regarding treatment.

Time of measurement	Treatment	AECT	*p* value	AE-QoL	*p* value	No. of Attacks	*p* value
T0 (pre-treatment)	LTP	5.9	0.039	61.6	0.3665	8.2	0.488
	OD	3.7		68.9		11.1	
T1 (3 months)	LTP	11.7	0.5706	38.7	0.3775	3.9	0.1429
	OD	12.1		30.8		3.1	
T2 (6 months)	LTP	12.4	0.881	38.6	0.6518	2.9	0.4155
	OD	12.1		33.5		2.7	
T3 (9 months)	LTP	13.1	0.8814	32.4	0.7943	2.4	0.7172
	OD	12.3		35.1		2.1	
T4 (12 months)	LTP	12.3	0.21	37.4	0.3671	1.4	0.4851
	OD	13.2		28.9		1.4	

LTP, long term preventive therapy; OD, on demand treatment; AECT, angioedema control test; AE-QoL, angioedema quality of life.

## Discussions

The present study describes real-life clinical data regarding the benefits of the LTP with lanadelumab in Romanian HAE-C1INH patients. Being a relative new drug, with limited data on its long-term effectiveness and safety, our results can serve as additional information to the data published so far.

The evaluated patients were predominantly adults, females, with HAE type 1, and most of them used only OD therapy. Lanadelumab treatment was assessed during one year period using three parameters: AECT, AE-QoL and attacks frequency. These tools were completed every three months assuring a close monitoring of these patients.

Adherence to lanadelumab treatment was excellent, with no missed doses (each dose was recorded and verified) through the evaluation period. Although all participants reported a local, injection site reaction (redness, swelling, pain) as adverse event, with the exception of one patient who experienced moderate pain during the first two administrations, all side effects were mild in intensity. No issues with tolerability were observed throughout the study duration.

At pre-treatment evaluation (T0), patient showed a high frequency of attacks (mean score: 10.0), significant impairment of quality of life (total score: 66.1), and uncontrolled disease (AECT score: 4.5).

Comparable data were obtained in a survey conducted by our Center using the Hereditary Angioedema Quality of Life Questionnaire (HAE-QoL), completed by 94 patients with HAE-C1INH during the second year of the COVID-19 pandemic. The survey recorded a total HAE-QoL score of 78 (range: 25–135) and a mean attack rate of 13.8, assessed in a subgroup of 30 patients (36). Additional evidence of suboptimal disease control was provided by a separate survey conducted by our Center in March 2022, involving 56 HAE-C1INH patients, which showed mean AECT scores of 6.9 and 7.0 for the 3-month and 1-month recall versions, respectively (30).

These data indicate an important burden of the disease and an insufficient control with the treatments currently used in HAE patients from Romania.

Initiation of LTP with lanadelumab resulted in statistically significant improvements (*p* < 0.0001) across all three evaluated parameters at the T1 assessment: AECT score (12.0), AE-QoL score (35.1), and attack frequency (3.4). These improvements were sustained at T2, T3, and T4 visits, with no statistically significant differences observed between these time points for AECT and AE-QoL (Figures 2–4).

Regarding attacks frequency, the initial reduction observed between T0 and T1 was both statistically significant (*p* < 0.0001) and clinically meaningful, representing a substantial decrease of approximately 66%. Although this reduction was notable, it was somewhat lower than those reported in the Hereditary angioEdema Long-term Prophylaxis Study (HELP, NCT02586805) (37) which demonstrated an 87.0% reduction and its open-label extension part (HELP OLE, NCT02741596) (38) which reported reductions of 82.0% in non-rollover patients and 92.4% in rollover patients.

In contrast, subsequent intervals demonstrated smaller reductions. From T1 to T2, the decrease was not statistically significant (*p* = 0.104) and corresponded to a 17.7% reduction. However, statistically significant decreases were again observed between T2 and T3 (*p* = 0.0273) and between T3 and T4 (*p* = 0.0049), with reductions of 24.1% and 36.4%, respectively. This can be explained by the fact that in some patients the number of attacks decreased progressively over time, with several individuals becoming asymptomatic by the T3 or T4 evaluations.

Our findings demonstrate the rapid onset of lanadelumab's effectiveness shortly after treatment initiation, and its sustained efficacy over time. Similar results have been reported by other groups in Germany (39–41) Poland (42), the United States (43), and Canada (44), confirming both early (within the first month) and long-term (6-, 12-, and 48-month) effectiveness.

Based on our findings, routine follow-up every three months appears unnecessary when lanadelumab is initiated as a LTP therapy. We propose a structured follow-up schedule: an initial assessment at 3 months, a second visit at 6 months to evaluate the possibility of dose reduction to 300 mg/month in asymptomatic patients, and subsequent annual follow-up.

In our study, improvement was observed regardless of whether patients were receiving only OD treatment or were on LTP with intravenous pdC1-INH at a dose of 2 × 1,000 IU per week. The degree of amelioration was similar between the two groups; however, a statistically significant difference (*p* < 0.05) was noted at the T0 assessment in AECT scores (3.7 vs. 5.9), but not in AE-QoL scores (38.9 vs. 61.6) or attack rates (11.1 vs. 8.2) (Table 4). Similar findings were reported in research conducted by Buttgereit et al. (39) who observed AECT score improvement in HAE patients treated with lanadelumab, without statistically significant pre-treatment differences between those receiving only OD therapy and those on LTP with pdC1-INH. Collectively, these results suggest that lanadelumab may be more effective than intravenous pdC1-INH for long-term prophylaxis. This finding is further supported by data from the PATCH study (44), an indirect treatment comparison demonstrating that patients treated with lanadelumab experienced less than half the number of attacks compared to those receiving intravenous pdC1-INH.

A reduction in attack frequency was also reported by Sánchez-Machín et al., who observed a similar trend in four patients with HAE-C1-INH. In their study, patients previously receiving long-term prophylaxis with pdC1-INH (three patients) or Berotralstat combined with danazol (one patient) experienced a decrease in attack frequency after switching to lanadelumab treatment (45).

Complete disease control, defined as an AECT score of 16, was achieved in five patients at the T1 assessment; by the T4 visit, one additional patient had reached this level, bringing the total to six, one from the prior LTP group and five from the OD treatment group. All six patients became symptom-free starting at T1. At the T4 visit, an additional 15 patients had AECT scores between 10 and 15, indicating controlled disease. In two cases, an AECT score of 10 was observed only at the T2 visit, with values below 10 at T1, T3, and T4. One patient consistently presented with an AECT score below 10 at all visits and did not achieve the minimal clinically important difference (MCID) of three points between assessments. Interestingly, this patient's subjective report contradicted the AECT findings; she reported feeling significantly better with lanadelumab treatment, citing reduced attack frequency (from 15 at T0 to 9 at T4), decreased severity, and, most notably a change in attack location, with monthly episodes of upper airway edema decreasing to one episode every three months. In this same patient, a single episode of respiratory tract swelling occurred after a dental extraction performed without pre-procedural prophylaxis.

Upper airway edema occurred in two additional patients during lanadelumab treatment and was successfully managed with self-administered icatibant. Prior to initiating LTP with lanadelumab, 19 patients (79.2%) had a history of upper airway edema. These findings align with those reported by Magerl et al. (44), who demonstrated that lanadelumab reduced the incidence of potentially life-threatening laryngeal attacks fivefold compared to LTP with pdC1-INH.

In our cohort, lanadelumab achieved a symptom-free status in seven patients (29.2%) after the first dose, and in an additional four patients within the first three months of treatment (without swelling episodes after the first dose of lanadelumab and subsequent injections within the first three months of treatment respectively). By the T4 assessment, 14 patients (58.3%), equally distributed between males and females remained attack-free, suggesting comparable efficacy across sexes. Although HAE is generally more severe in females, as also reflected in our baseline data (T0), statistically significant differences (*p* < 0.05) were observed in total AE-QoL scores (73.79 vs. 55.40 in females vs. males), as well as in the functional domain (76.57 vs. 58.20) and the fear/shame domain (83.07 vs. 62.60). Notably, these sex-related disparities were no longer present starting at the T1 assessment and remained absent at T4 (Table 3).

Quality of life improved significantly in patients treated with lanadelumab, as demonstrated by a statistically significant decrease in AE-QoL total scores between T0 and T1 (*p* < 0.0001). Although improvement continued at subsequent time points, it was modest, and remained within this range throughout the study period (Figure 4).

A similar pattern was observed across domain-specific AE-QoL scores: significant improvements were recorded at T1 for all domains (*p* < 0.0001), but no statistically significant changes were observed at T2, T3, or T4 (Table 2).

Notably, some domains showed slight deterioration at specific intervals. A minor decline in functioning was observed between T3 and T4, while both the fatigue/mood domain and the total score decreased slightly from T1 to T2. The most pronounced reduction occurred in the nutrition domain between T1 and T2. In contrast, the fear/shame domain showed continuous improvement across all visits, with no decline observed These findings suggest that each domain contributed relatively equally to the persistence of moderate impairment.

Further investigation is warranted to identify the underlying factors responsible for the sustained moderate level of quality-of-life impairment despite clinical improvements.

Regarding treatment effect classification, a “no effect” AE-QoL category (score 0–23) was observed in eight patients (33.4%) at T1 and nine (37.5%) at T4. A “small effect” category (score 24–38) was recorded in seven patients (29.2%) at T1 and six (25%) at T4.

### Study limitations and further studies

A strength of this study lies in the use of validated tools adapted for the Romanian language. However, a limitation is that these instruments are not specific to hereditary angioedema (HAE). Additionally, the simultaneous administration of two questionnaires may introduce potential bias, particularly given the different recall periods: the AECT assesses disease control over the previous three months, while the AE-QoL reflects quality of life over the past four weeks. Another possible source of bias is the online administration of the questionnaires in many cases, which may have influenced participant responses.

As more patients are enrolled in the lanadelumab treatment program, we anticipate that further studies will be conducted to expand the evidence base regarding the long-term efficacy and safety of this therapy.

## Conclusions

Our study demonstrated that lanadelumab is an effective and well-tolerated treatment for patients with hereditary angioedema (HAE) in Romania. This therapy provided adequate disease control and significantly improved the patients “quality of life within a relatively short time after initiation, with sustained benefits observed over the one-year treatment period. Regular evaluations at relatively short intervals by the availability and ease of administration of validated questionnaires serve as a useful tool for clinicians in the comprehensive assessment of HAE patients and offer a valuable means of monitoring treatment effectiveness.”

## Data Availability

The original contributions presented in the study are included in the article/Supplementary Material, further inquiries can be directed to the corresponding author.

## References

[1] Banerji A Kimberly HD Brown TM Hollis K Hunter SM Long J Patient-reported burden of hereditary angioedema: findings from a patient survey in the United States. Ann Allergy Asthma Immunol. (2020) 124:600–7. 10.1016/j.anai.2020.02.018 32169514

[2] Bork K Anderson JT Caballero T Craig T Johnston DT Li HH Assessment and management of disease burden and quality of life in patients with hereditary angioedema: a consensus report. Allergy Asthma Clin Immunol. (2021) 17:40. 10.1186/s13223-021-00537-2 33875020 PMC8056543

[3] Zuraw B. Hereditary angioedema. N Engl J Med. (2008) 359(10):1027–36. 10.1056/NEJMcp0803977 18768946

[4] Cicardi M Bork K Caballero T Craig T Li HH Longhurst H Evidence-based recommendations for the therapeutic management of angioedema owing to hereditary C1 inhibitor deficiency: consensus report of an international working group. Allergy. (2012) 67(2):147–57. 10.1111/j.1398-9995.2011.02751.x 22126399

[5] Balla ZS Ignac B Varga L Kohalmi KV Farkas H. How angioedema quality of life questionnaire can help physicians in treating C1-inhibitor deficient patients? Clin Rev Allergy Immunol. (2021) 61:50–9. 10.1007/s12016-021-08850-9 33660212 PMC8282561

[6] Zuraw BL Christiansen SC. HAE pathophysiology and underlying mechanisms. Clin Rev Allergy Immunol. (2016) 51(2):216–29. 10.1007/s12016-016-8561-8 27459852

[7] Gower RG Busse P Aygören Pürsün E Barakat AJ Caballero T Davis-Lorton M Hereditary angioedema caused by C1-esterase inhibitor deficiency: a literature-based analysis and clinical commentary on prophylaxis treatment strategies. WAO J. (2011) 4(Suppl 2):9–21. 10.1097/1939-4551-4-S2-S9 PMC3666183 23283143

[8] Bork K Hardt J Witzke G. Fatal laryngeal attacks and mortality in hereditary angioedema due to C1-INH deficiency. J Allergy Clin Immunol. (2012) 130(3):692–7. 10.1016/j.jaci.2012.05.055 22841766

[9] Bork K Machnig T Wulff K Witzke G Prusty S Hardt J. Clinical features of genetically characterized types of hereditary angioedema with normal C1 inhibitor: a systematic review of qualitative evidence. Orphanet J Rare Dis. (2020) 15:289. 10.1186/s13023-020-01570-x 33059692 PMC7559394

[10] Dewald G Bork K. Missense mutations in the coagulation factor XII (hageman factor) gene in hereditary angioedema with normal C1inhibitor. Biochem Biophys Res Commun. (2006) 343(4):1286–9. 10.1016/j.bbrc.2006.03.092 16638441

[11] Bafunno V Firinu D D’Apolito M Cordisco G Loffredo S Leccese A Mutation of the angiopoietin-1 gene (ANGPT1) associates with a new type of hereditary angioedema. J Allergy Clin Immunol. (2018) 141(3):1009–17. 28601681 10.1016/j.jaci.2017.05.020

[12] Bork K Wulff K Steinmuller-Magin L Braenne I Staubach-Renz P Witzke G Hereditary angioedema with a mutation in the plasminogen gene. Allergy. (2018) 73(2):442–50. 10.1111/all.13270 28795768

[13] Bork K Wulff K Rossmann H Steinmuller-Magin L Braenne I Witzke G Hereditary angioedema cosegregating with a novel kininogen 1gene mutation changing the N-terminal cleavage site of bradykinin. Allergy. (2019) 74(12):2479–81. 31087670 10.1111/all.13869

[14] Bork K Wulff K Hardt J Witzke G Staubach P. Hereditary angioedema caused by missense mutations in the factor XII gene: clinical features, trigger factors, and therapy. J Allergy Clin Immunol. (2009) 124(1):129–34. 10.1016/j.jaci.2009.03.038 19477491

[15] Ariano A D’Apolito M Bova M Bellanti F Loffredo S D’Andrea G A myoferlin gain-of-function variant associates with a new type of hereditary angioedema. Allergy. (2020) 75(11):2989–92. 10.1111/all.14454 32542751

[16] Bork K Wulff K Möhl BS Steinmüller-Magin L Witzke G Hardt J Novel hereditary angioedema linked with a heparan sulfate 3-O-sulfotransferase 6 gene mutation. J Allergy Clin Immunol. (2021) 148:1041–8. 10.1016/j.jaci.2021.01.011 33508266

[17] Denis V Parsopoulou F Martin L Gaboriuad C Demongeot J Loules G. Hereditary angioedema with normal C1 inhibitor associated with carboxypeptidase N deficiency. J Allergy Clin Immunol Glob. (2024 Feb 1) 3(2):100223. 10.1016/j.jacig.2024.100223 38445235 PMC10912455

[18] D’Apolito M Santacroce R Vazquez DO Cordisco G Fantini CA D’Andrea G DAB2IP associates with hereditary angioedema: insights into the role of VEGF signaling in HAE pathophysiology. J Allergy Clin Immunol. (2024) 154(3):698–706. 10.1016/j.jaci.2024.05.017 38823490

[19] Maurer M Magerl M Betschel S Aberer W Ansotegiu IJ Aygören Pürsün E The international WAO/EAACI guideline for the management of hereditary angioedema—the 2021 revision and update. Allergy. (2022) 77:1961–90. 10.1111/all.15214 35006617

[20] Aabom A Andersen KE Perez-Fernandez E Caballero T Bygum A. Health related quality of life in Danish patients with hereditary angioedema. Acta Derm Venereol. (2015) 95(2):225–6. 24603953 10.2340/00015555-1835

[21] Arce-Ayala YM Diaz-Algorri Y Craig T Ramos-Romey C. Clinical profile and quality of life of Puerto Ricans with hereditary angioedema. Allergy Asthma Proc. (2019) 40(2):103–10. 10.2500/aap.2019.40.4200 30819279

[22] Bouillet L Launay D Fain O Boccon-Gibod I Laurent J Martin L Hereditary angioedema with C1 inhibitor deficiency: clinical presentation and quality of life of 193 French patients. Ann Allergy Asthma Immunol. (2013) 111(4):290–4. 10.1016/j.anai.2013.07.012 24054366

[23] Gomide MA Toledo E Valle SO Campos RA França AT Gomez NP Hereditary angioedema: quality of life in Brazilian patients. Clinics (Sao Paulo). (2013) 68(1):81–3. 10.6061/clinics/2013(01)OA13 23420162 PMC3552471

[24] Jindal NL Harniman E Prior N Perez-Fernandez E Caballero T Betschel S. Hereditary angioedema: health-related quality of life in Canadian patients as measured by the SF-36. Allergy Asthma Clin Immunol. (2017) 13:4. 10.1186/s13223-016-0176-3 28115964 PMC5244704

[25] Liu S Wang X Xu Y Xu Q Zhi Y. Health-related quality of life and its risk factors in Chinese hereditary angioedema patients. Orphanet J Rare Dis. (2019) 14(1):191. 10.1186/s13023-019-1159-5 31395105 PMC6686410

[26] Nordenfelt P Nilsson M Lindfors A Wahlgren CF Björkander J. Health-related quality of life in relation to disease activity in adults with hereditary angioedema in Sweden. Allergy Asthma Proc. (2017) 38(6):447–55. 10.2500/aap.2017.38.4087 28855002

[27] Caballero T Prior N. Burden of illness and quality-of-life measures in angioedema conditions. Immunol Allergy Clin North Am. (2017) 37(3):597–616. 10.1016/j.iac.2017.04.005 28687112

[28] Gabos G Nadasan V Mihaly E Dobru D. Hereditary angioedema due to C1-inhibitor deficiency in Romania: first national study, diagnostic and treatment challenges. Iran J Immunol. (2020) 17(3):226–35. 10.22034/iji.2020.85416.1709 32996899

[29] Betschel S Badiou J Binkley K Borici-Mazi R Hébert J Kanani A The international/Canadian hereditary angioedema guideline. Allergy Asthma Clin Immunol. (2019) 15:72. 10.1186/s13223-019-0376-8 31788005 PMC6878678

[30] Bara NA Nadasan I Nadasan V Deleanu D. Assessing the control of the disease on current treatments available in Romania for hereditary angioedema patients. Acta Marisiensis Ser Med. (2024) 70(2):1. 10.2478/amma-2024-0009

[31] Weller K Donoso T Magerl M Aygören-Pürsün E Staubach P Martinez-Saguer I Validation of the angioedema control test (AECT)-A patient-reported outcome instrument for assessing angioedema control. J Allergy Clin Immunol Pract. (2020) 8(6):2050–e4. 10.1016/j.jaip.2020.02.038 32173507

[32] Weller K Groffik A Magerl M Tohme N Martus P Krause K Development and construct validation of the angioedema quality of lifequestionnaire. Allergy. (2012) 67(10):1289–98. 10.1111/all.12007 22913638

[33] Weller K Donoso T Magerl M Aygören-Pürsün E Staubach P Martinez-Saguer I Development of the angioedema control test-A patient-reported outcome measure that assesses disease control in patients with recurrent angioedema. Allergy. (2020) 75(5):1165–77. 10.1111/all.14144 31815297

[34] Fijen LM Vera C Buttgereit T Bonnekoh H Maurer M Magerl M Sensitivity to change and minimal clinically important difference of the angioedema control test. Clin Transl Allergy. (2023) 13:e12295. 10.1002/clt2.12295 37746798 PMC10472988

[35] Weller K Magerl M Peveling-Oberhag A Martus P Staubach P Maurer M. The angioedema quality of life questionnaire (AE-QoL)—assessment of sensitivity to change and minimal clinically important difference. Allergy. (2016) 71(8):1203–9. 27038109 10.1111/all.12900

[36] Nadasan V Nadasan A Borka Balas R Bara NA. A cross-sectional study of quality of life in patients enrolled in the Romanian hereditary angioedema registry. Cureus. (2024) 16:e51959. 10.7759/cureus.51959 38196989 PMC10776050

[37] Banerji A Riedl MA Bernstein JA Cicardi M Longhurst HJ Zuraw BL Effect of lanadelumab compared with placebo on prevention of hereditary angioedema attacks. JAMA. (2018) 320(20):2108–21. 10.1001/jama.2018.16773 30480729 PMC6583584

[38] Banerji A Bernstein JA Johnston DT Lumry WR Magerl M Maurer M Long-term prevention of hereditary angioedema attacks with lanadelumab: the HELP OLE study. Allergy. (2022) 77(3):979–90. 10.1111/all.15011 34287942 PMC9292251

[39] Buttgereit T Vera C Weller K Gutsche A Grekowitz EM Aykanat S Lanadelumab efficacy, safety, and injection interval extension in HAE: a real-life study. J Allergy Clin Immunol Pract. (2021) 9(10):3744–51. 10.1016/j.jaip.2021.04.072 34023564

[40] Magerl M Bouillet L Martinez-Saguer I Gavini F Bent-Ennakhil N Sayegh L Real-world effectiveness of lanadelumab in hereditary angioedema: multicountry INTEGRATED observational study. J Allergy Clin Immunol Pract. (2025) 13(2):378–87. 39701274 10.1016/j.jaip.2024.12.008

[41] Hahn J Trainotti S Wigand MC Schuler PJ Hoffmann TK Greve J. Prospective analysis in patients with HAE under prophylaxis with lanadelumab: a real-life experience. J Drugs Dermatol. (2020) 19:978–83. 10.36849/JDD.2020.5269 33026762

[42] Kucharczyk A Matuszewski T Kurowski M Juchacz A Tomasial-Lozowska M Trebas-Pietras E Real-world treatment outcomes of lanadelumab in the prevention of hereditary angioedema attacks: an interim analysis of a Polish, prospective, multicenter, observational study (CHOPIN). J Allergy CLIN Immunol. (2024) 153(2):AB9.

[43] Lumry WR Weller K Magerl M Banerji A Longhurst HJ Riedl MA Impact of lanadelumab on health-related quality of life in patients with hereditary angioedema in the HELP study. Allergy. (2021) 76:1188–98. 10.1111/all.14680 33258114 PMC8247292

[44] Magerl M Schiffhorst G Fanter L Müller G Hirche C Berkemeier F Patient-level indirect treatment comparison of lanadelumab versus pdC1-INH i.v. in hereditary angioedema patients: PATCH study. Allergy. (2024) 79(1):215–24. 10.1111/all.15861 37641968

[45] Sánchez-Machín I González-Pérez R Mederos-Luis E García-Gil S Poza-Guedes P. Real-world outcomes and healthcare utilization of lanadelumab in Spain: insights from first cohort of difficult-to-treat hereditary angioedema cases. Allergies. (2025) 5(1):3. 10.3390/allergies5010003

